# MicroNAS for memory and latency constrained hardware aware neural architecture search in time series classification on microcontrollers

**DOI:** 10.1038/s41598-025-90764-z

**Published:** 2025-03-04

**Authors:** Tobias King, Yexu Zhou, Tobias Röddiger, Michael Beigl

**Affiliations:** Karlsruhe Institute of Technology, TECO, 76131 Karlsruhe, Germany

**Keywords:** Computer science, Information technology

## Abstract

The use and research of neural networks on very small processor systems are currently still limited. One of the main reasons is that the design of microcontroller-architecture-aware ML models that take into account user-defined constraints on memory consumption and run-time are very difficult to implement. Therefore, we adapt the concept of differentiable neural architecture search (DNAS) to solve the time series classification problem on resource-constrained microcontrollers (MCUs). This paper explores and demonstrates for the first time that this problem can be solved using Neural Architecture Search (NAS). The key of our specific hardware-aware approach, MicroNAS, is an integration of a DNAS approach, Latency Lookup Tables, Dynamic Convolutions and a novel search space specifically designed for time series classification on MCUs. The resulting system is hardware-aware and can generate neural network architectures that satisfy user-defined limits on execution latency and peak memory consumption. To support our findings, we evaluate MicroNAS under different latency and peak memory constraints. The experiments highlight the ability of MicroNAS to find trade-offs between latency and classification performance across all dataset and microcontroller combinations. As an example, on the UCI-HAR dataset, MicroNAS achieves an accuracy of 94.62% when allowed 25 ms and 98.86% when allowed 50 ms when running on the Nucleo-L552ZE-Q. The much more powerful Arduino Portenta, on the other hand, achieves an accuracy of 95.88% with an allowance of 3 ms and 99.37% when allowed 25 ms displaying the ability of MicroNAS to adapt to different microcontrollers. MicroNAS is also able to find architectures which perform similarly to state-of-the-art systems designed to run on desktop computers (99.62% vs. 99.65% accuracy on the UCI-HAR dataset and 97.83% vs. 97.46% accuracy on the SkodaR dataset).

## Introduction

MCUs are small, low-power computing systems that can be found in a wide range of devices, including medical equipment, consumer electronics, wearables and many more. Deploying machine learning models directly on microcontrollers enables applications such as predictive maintenance^1^, human activity recognition^2^ or health monitoring^3^ to be always available without network connectivity while ensuring privacy^4^. Many of these devices utilize sensors, such as, accelerometers, gyroscopes and more which generate time series data^5^.

The combination of sensors and microprocessors embedded in smart sensors creates the opportunity for offline, on-device data analysis which allows these devices to operate in privacy-critical, real-time and autonomous systems^6^. Due to the limited hardware of typical MCUs (e.g. 64 KB SRAM, 64 MHz CPU clock), it is not possible to run state-of-the-art time series classification architectures such as InceptionTime^7^ or DeepConvLSTM^8^ on these devices. Our focus is on low-power MCUs that are widely used in resource-constrained environments such as sensor nodes (e.g. Cortex-M4 equipped MCUs), rather than more powerful microprocessors found in smartphones and single-board computers with significantly more computational resources.

A common solution to deal with the limited resources of microcontrollers is to send the raw data to a server in the cloud, where state-of-the-art models can be executed and then transmit the result back to the microcontroller. For many reasons, this approach is not sustainable: network communication introduces uncertain latencies, preventing its use in real-time applications or scenarios where networking is unavailable, and processing data on external servers poses a privacy risk. In addition, network communication is expensive for microcontrollers in terms of energy consumption. One promising approach is to deploy neural networks directly on microcontrollers. Recent research^9–11^ has demonstrated that neural networks can outperform traditional classifiers in resource-constrained environments. Unlike traditional methods, neural networks offer end-to-end learning capabilities that eliminate the need for manual feature extraction. Moreover, their architectural flexibility enables the design of specialized neural network models precisely tailored to specific use cases, maximizing performance within tight computational and memory constraints. The design of these specific neural networks is often done by domain experts with knowledge in the field of machine learning and is an error-prone and time-consuming process^12^. To automate this design process, neural architecture search (NAS) can be applied to find suitable neural network architectures for specific use-cases. Existing state-of-the-art NAS-systems focus on generating neural network architectures for image classification^13–15^. Hardware-aware NAS systems (HW-NAS) which optimize classification accuracy and hardware utilization, have also been implemented for image classification^16–18^. However, existing HW-NAS systems are not adapted to the time series classification task and utilize latency estimation methods that are not precise enough for highly constrained microcontrollers^15–17^.

To apply HW-NAS to time series classification, two main challenges need to be overcome. First, the shape of time series data differs fundamentally from image data which requires an adaptation of the search space. We solve this problem by introducing a novel, two stage search space, in which first Time-Reduce cells extract temporal context and in a second step, Sensor-Fusion cells allow for cross-channel interaction^19^. Depending on the window-size and the number of sensor-channels, we vary the number of cells in the search space to cover a wide range of time series datasets. Second, to be able to adhere to the resource constraints of MCUs and select the best architecture, a fine granular search space in combination with precise execution latency predictions is required. If the search space is too coarse, it may not be possible to find optimal architectures for the given task that still satisfy user imposed limits on the execution latency and peak memory consumption. Similarly, imprecise execution latency estimations make it impossible to determine when the maximum allowed execution latency is exceeded. We utilize a masking convolution approach adapted from^15^ to create a fine granular search space by varying the number of filters in convolutional layers. To precisely estimate the execution latency of architectures in the search space, we employ a latency lookup table based approach^14^. FBNetV2^15^ employs a technique called effective-shape-propagation in order to estimate the execution latency of architectures. This approach is not compatible with the lookup-table based approach but we overcome this limitation by linking these two techniques with an interpolation schema. In summary, this paper makes the following contributions:

1. MicroNAS; the first hardware-aware neural architecture search (HW-NAS) system for time series classification tasks on embedded microcontrollers.
2. Introduction of a time series classification specific search space suitable for datasets with varying window sizes and number of sensors. The search space contains two searchable cells that extract temporal information and allow for cross-channel interaction respectively.
3. An automatic characterization method to calculate neural architecture execution latencies for microcontrollers based on a lookup table with an average error of $\approx \pm {1.59}\,\textrm{ms,}$, showing that this approach outperforms proxy latency metrics ($\approx \pm {15.57}\,\textrm{ms}$).

**Fig. 1:**
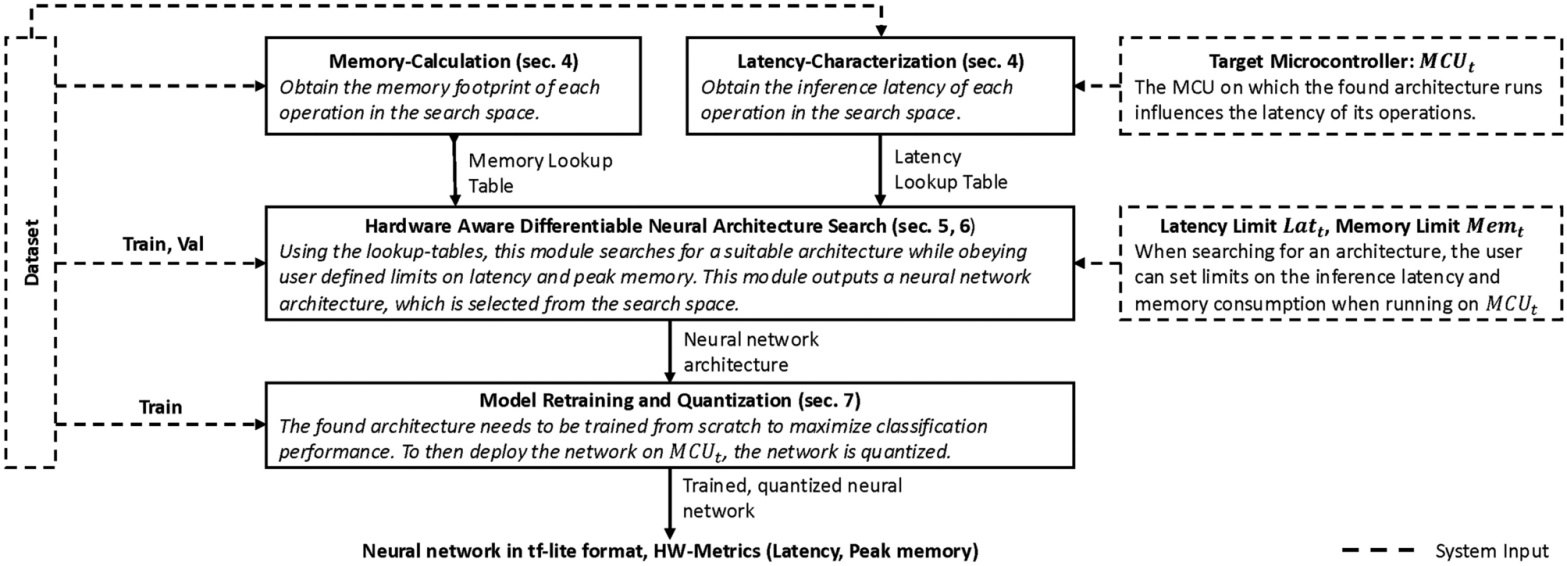
MicroNAS requires the dataset to be split into three different sets which are used at different stages in the pipeline. The user specifies the dataset to be used, the target MCU ($MCU_t$) and the maximum allowed hardware utilization in terms of execution latency ($Lat_t$) and peak memory consumption ($Mem_t$). Output of the system is a corresponding neural network in the tf-lite format.

## Background and related work

We first summarize time series classification using deep learning approaches and then introduce existing state-of-the-art neural architecture search systems.

### Time series classification

In the recent past, deep learning-based approaches have been used successfully for time series classification. Systems such as InceptionTime^7^ and multi-scale CNNs^20^ feature CNN based architectures to aggregate temporal context on multiple scales. Other options include the use of RNNs or hybrid models consisting of both CNN and RNN layers^8,21^. However, deploying these models on microcontrollers (MCUs) remains challenging due to their computational and memory constraints. To address this issue, lightweight neural networks optimized for MCU-based time series classification have been proposed^22,23^. Recent methods, such as TinyLSTMs^24^, TinyHAR^19^ and MLP-HAR^25^ focus on designing efficient architectures that balance accuracy and resource constraints. Inspired by the existing CNN system architectures, we develop our NAS search space. This space features searchable cells that are optimized for MCUs while preserving the hierarchical structures typically found in CNN-based time series classification models.

### Neural architecture search

Early neural architecture search systems (NAS)^26^ formulate the search as a reinforcement learning problem. While this approach produces novel, well-performing architectures, the search takes long as each iteration of the REINFORCE algorithm requires training a neural network until convergence with no weight sharing between architectures. To overcome this issue, super-networks have been introduced as search spaces, where each architecture exists as a subgraph, allowing for shared weights among them^13,27^. Systems like^28^ and^27^ show, that training the super-network is enough to emulate any architecture in the search space. Darts extends this idea by introducing Differentiable Neural Architecture Search (DNAS)^13^. DNAS utilizes a relaxation schema to make the search continuous, differentiable and, therefore more resource efficient. In DNAS, the search space is also defined by a super-network, in which a layer has not one but multiple operations. The layer output *l* is then computed as a convex combination of the outputs of the operations *o* scaled by the architectural weights $\alpha:$ $l = \sum _{i} o_i * \alpha _i$. During architecture search, the regular neural network weights and the architectural weights are jointly optimized using gradient descent. This allows for a structured and more efficient search. After training is complete, the architectural weights are used to identify the selected architecture. Several works have extended DNAS to constrained environments, such as TinyML and embedded systems^29–32^. Due to its efficiency, we use DNAS as the basis for the search algorithm of MicroNAS.

Recently, NAS has been extended to be hardware aware (HW-NAS). Systems in this category not only optimize for classical performance metrics such as accuracy or precision but also for hardware-specific metrics such as execution latency, peak memory and energy consumption^33,34^. Optimizing the hardware utilization is especially important when targeting microcontrollers, as these devices are typically severely resource-constraint. Therefore, during architecture search, the search algorithm needs to be able to estimate relevant hardware metrics for arbitrary architectures. For the peak memory consumption, analytical estimation can be used for precise calculation. In contrast, for the execution latency, many approaches exist^33^. Real-time latency measurements on the target hardware during architecture search provide precise measurements but prolong the search drastically^33,35^. Another common and much faster approach is to use the number of FLOPs or similar metrics as a proxy for the execution latency^14,16,17^. While the authors of MicroNets^16^ and $\mu$Nas^17^ claim the number of operations in a model is a good proxy for the execution latency when targeting MCUs, others^36^ argue that this is not the case. A middle ground between the slow but precise on-device measurements during search and the fast but imprecise latency estimations using the number of operations, are lookup tables^33,37^. With the lookup table approach, operations in the search space are executed on the MCU once and can then be efficiently used during search time.

Most NAS approaches for time series tasks focus on classification^38^ or forecasting^39^, but they do not optimize for MCUs. In contrast, HW-NAS methods such as MicroNets^16^ and $\mu$NAS^17^ optimize for constrained hardware but do not address time series classification. The proposed system bridges this gap by integrating hardware-aware NAS techniques, including DNAS and lookup-table-based latency estimation, while featuring a specialized search space designed for time series classification on MCUs. By combining state-of-the-art NAS techniques with time series-specific architectures, MicroNAS provides a comprehensive solution for MCU-compatible time series classification models that balance accuracy, efficiency, and resource constraints.

## System overview

The system overview of MicroNAS can be seen in Fig. 1. The input to the system consists of a time series dataset, an MCU to use ($MCU_t$) and user-defined limits on the execution latency ($Lat_t$) and peak memory consumption ($Mem_t$). In a first step, the hardware utilization of each operator in the search space is obtained in an operation called characterization, shown in “Latency and peak memory estimation” section. After characterization, HW-NAS is executed where a DNAS approach (“Search algorithm” section) is utilized to select a suitable architecture from our search space (“Search space” section) for the dataset and $MCU_t$ combination. The found architecture is then extracted from the search space and retrained from scratch using quantization aware training to maximize classification performance (“Model retraining and quantization” section). Finally, the trained, $int\_8$ quantized neural network is converted to the tf-lite format and can now be deployed on $MCU_t$.

## Latency and peak memory estimation

For MicroNAS to find architectures that obey user-defined limits on the execution latency and peak-memory consumption, it is necessary to estimate the actual execution latency and peak-memory consumption of individual architectures in the search space. To improve on flops-based proxy metrics for the execution latency, we introduce a lookup-table-based approach. The peak memory consumption is estimated analytically, following the approach of^16,17^.

### Latency characterization

**Fig. 2:**
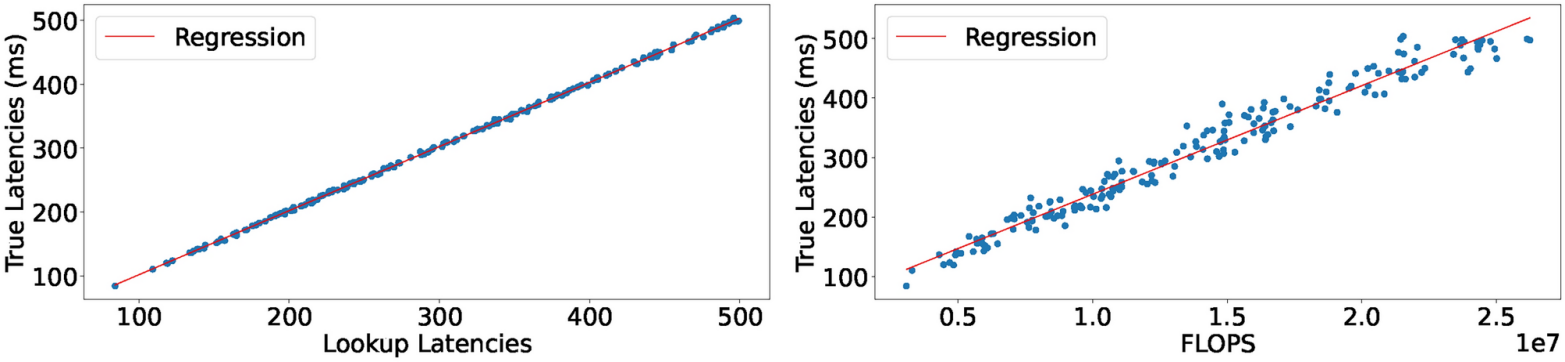
Execution latency of whole architectures from our search space. Left: Our lookup-table latency approach. MAE: 1.59 ms, $R^{2}$: 0.9997. Right: Flops based estimate: MAE: 15.57 ms, $R^{2}$: 0.9678.

When calculating the execution latency of neural network architectures, the literature proposes to use the number of operations in an architecture as a proxy metric^16,17,40^. We argue for a lookup table based approach, in which we obtain the execution latency of each operator in our search space by executing it on the actual MCU. From this information, we can calculate the execution latency for arbitrary architectures in the search space. To determine the viability of this approach, we conduct our own experiment in which we compare our latency lookup table approach with a FLOPs-based proxy metric as seen in Fig. 2. We executed the experiment using $int\_8$ quantized networks on the Nucleo-L552ZE-Q where we measured the actual execution latency by using the internal CPU-cycle counter on ARM Cortex processors. Results can be seen in Fig. 2. Our lookup table approach achieves an $R^2$-score of 0.9997 with a mean absolute error of 1.59 ms. The FLOPs based latency estimation achieves an $R^2$-score of 0.9678 and a mean absolute error of 15.57 ms. Therefore, we can conclude, that the lookup table approach is able to outperform the flops-based latency estimation approach.

## Search space

To accommodate the time series classification task, we introduce a novel, MCU-tailored search space consisting of two types of architecture-searchable cells. This search space is defined by a super-network, built from a linear stack of architecture-searchable cells. First, Time-Reduce cells are utilized to extract temporal context from the incoming time series. In a second step, Sensor-Fusion cells allow for cross-channel interactions where information from multiple sensors can be fused. This two-step process is a common approach in the domain of time series classification^8,19,20,41,42^. Each of the searchable cells is hardware aware and therefore output their hardware metrics, including execution latency $Lat(\alpha , MCU_t)$ and peak memory consumption $Mem(\alpha , MCU_t)$ which depend on the architectural weights $\alpha$ and $MCU_t$. Input to the search space are time series windows, represented by 2D-Tensors with dimensions $ts_l$ (window size) and $ts_s$ (Sensor channels). To adapt the search space dynamically to datasets with varying window sizes ($ts_l$) and number of sensor-channels ($ts_s$), the number of cells is adapted automatically. The number of Time-Reduce cells ($N_{TR}$) is calculated according to Eq. 1:

1 $$\begin{aligned} N_{TR} = \left\lfloor \log _2\left( \frac{ts_{l}}{ts_{ml}}\right) \right\rfloor \end{aligned}$$

$$ts_l$$$$ts_{ml}$$ the minimum. (1) and the number of Sensor-Fusion cells ($$N_{SF}$$) is calculated according to Eq. (2):

2 $$\begin{aligned} N_{SF} = \left\lfloor \log _2\left( \frac{ts_{s}}{ts_{ms}}\right) \right\rfloor \left( 1 + sf_{s}\right) \end{aligned}$$

**Fig. 3:**
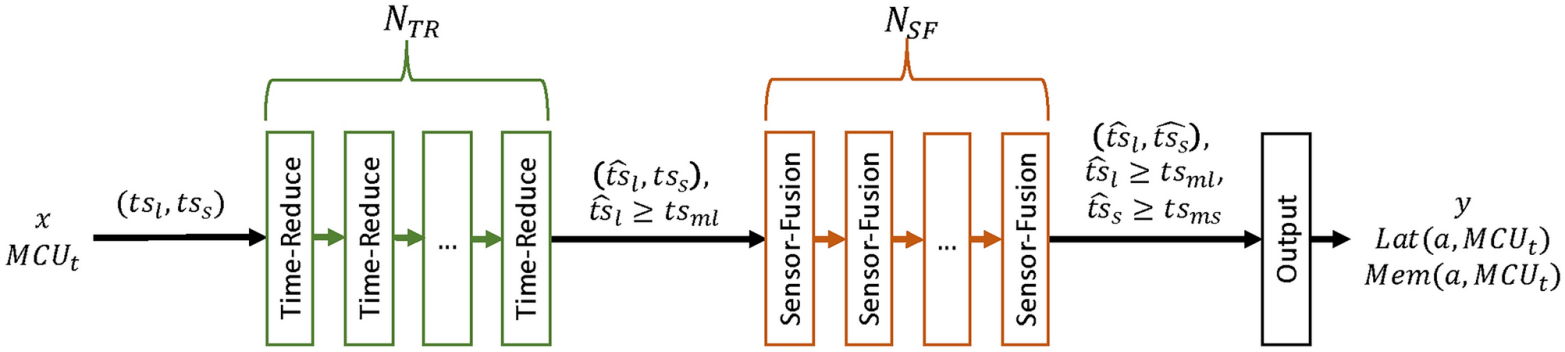
High-level overview over the search space. The raw, windowed time series *x* with shape $(ts_l, ts_s)$ is propagated though $N_{TR}$ Time-Reduce and $N_{SF}$ many Sensor-fusion cells. The resulting time series is then of shape $(\hat{ts}_l, \hat{ts}_s)$. Class probabilities *y* and hardware metrics are output by the Output cell at the end of the network.

$ts_{ml}$ is the minimum window size after the Time-Reduce cells while $ts_{ms}$ is the minimum size of the sensor-dimension after the Sensor-Fusion cells. The parameter $sf_s$ is user settable to increase the number of Sensor-Fusion cells which allows for deeper networks. The Sensor-Fusion cells can be configured with stride 1 or 2 while the number of cells with stride 2 is independent of the parameter $sf_s$. An overview of this search-space can be seen in Fig. 3. To improve stability during training, dropout layers with dropout factor 0.3 are placed between all cells (not shown in figure).

### Decision groups

In DNAS, each searchable cell contains two sets of weights: The regular neural network weights *w* as well as the architectural weights $\alpha$ indicating the architecture. These architectural weights are organized in decision groups. A decision group $\alpha _i$ is a collection of architectural weights $\alpha _{i,j}$, used to make one-out-of-many decisions. $\alpha _{i,j}$ denotes the *j*-th architectural weight in the *i*-th decision group. Each weight $\alpha _{i,j}$ in a decision group gates a path in the cell and therefore, one-hot encoded decision groups define the cell-architecture. During search-time, a pseudo probability-function is applied to the decision group, shown in Eq. (3).

3 $$\begin{aligned} \hat{\alpha }_{i,j} = pseudo\_prob(\alpha _{i,j}) \end{aligned}$$

After the search, each decision group will be one-hot encoded. This effectively discards all options which are assigned the zero value and therefore the final architecture is determined.

### Dynamic convolutions

A dynamic convolution^15^ is a convolution whose number of filters can be searched for efficiently by using weight sharing. We adapt this concept and couple it with an interpolation scheme to make it compatible with our latency lookup table. In a dynamic convolution, first a convolution with the maximum number of allowed filters $f_{max}$ is applied to the input *x*. The output of this convolution is then multiplied with a binary mask in the filter dimension. This mask is the weighted sum of several masks $m_i$, with architectural weights $\hat{\alpha }_{y,i}$:

4 $$\begin{aligned} y = conv(x) * \left( \sum _{i} {\hat{\alpha }_{y, i} * m_i}\right) \end{aligned}$$

**Fig. 4:**
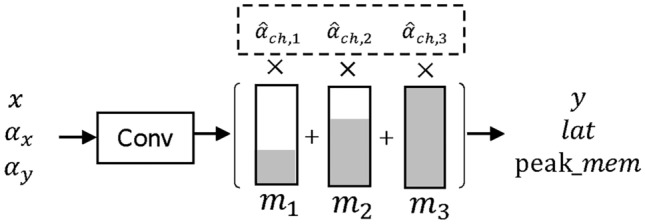
Dynamic convolution with three different options (e.g. $f_{max} = 24$, $g = 3$) for the number of filters. The binary masks ($m_i$) zero out certain filters in the output of the convolution. Grey areas are ones and white areas are zeros.

This formulation allows to efficiently search for the number of filters in a convolution by using the decision group $\alpha _{y}$. This process is displayed in Fig. 4. As the hardware utilization of a convolution also depends on the number of filters in the incoming time series, we need to take the decision group $\alpha _{x}$, responsible for the number of filters in the input into consideration. To reduce the cost of latency characterization, we introduce the granularity *g* with ($f_{max} \mod g == 0$). This parameter controls how many filters are disabled by one mask $m_i$. To characterize a dynamic convolution, we must execute all possible combinations of number of input and number of output filters on the $MCU_t$. The introduction of *g* reduces the number of possible combinations from $f_{max}^2$ to $(f_{max}/g)^2$ which significantly reduces characterization cost. Finally, execution latency and peak memory consumption for a dynamic convolution can be calculated with the interpolation schema according to Eq. (5). In the equation, the function *HW*(*x*, *y*) returns the execution latency and peak memory consumption for the dynamic convolution with *x* input and *y* output filters.

5 $$\begin{aligned} op_{hw} = \hat{\alpha }_{y}^T \cdot HW_{op} \cdot \hat{\alpha }_{x}, \quad HW_{op} = \begin{bmatrix} HW(g_x, g_y) & \dots & HW(\vert \alpha _{x}\vert g_x, g_y) \\ \vdots & \ddots & \vdots \\ HW(g_1, \vert \alpha _{y}\vert *g_y) & \dots & HW(\vert \alpha _{x}\vert g_x, \vert \alpha _{y}\vert g_y) \end{bmatrix} \end{aligned}$$

In the equation, $g_y$ denotes the granularity corresponding to the output of the convolution while $g_x$ corresponds to the granularity of the input. The same concept can be applied to the dynamic Add operations.

### Cells

To accommodate the time series classification task, two types of searchable cells are designed. The Time-Reduce cell aggregates information in the temporal domain while the Sensor-Fusion cell allows for cross-channel interaction. After each convolution in the architecture, a ReLU activation function is applied (not shown in graphics).

#### Time-reduce cell

**Fig. 5:**
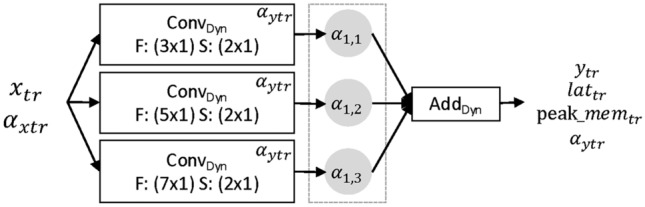
Time-Reduce cell. Contains two decision groups. $\alpha _1$ to choose a convolution and $\alpha _{ytr}$ to search for the number of filters. *F* is the filter size while *S* is the stride configuration.

The Time-Reduce cell shown in Fig. 5 aggregates local context by applying strided convolutions in the temporal dimension while leaving the sensor-dimension untouched (Filter size: $(\{3,5,7\} \times \textbf{1})$). This is done to reduce the window size of the propagated time series, to save on computational costs in subsequent cells but also to extract and fuse local initial features from the raw data^19^. The cell contains two decision groups. $\alpha _1$ to choose one of the convolutions and $\alpha _2$ to select the number of filters in that convolution. Input to this type of cell is a time series $x_{tr}$ of shape $(t_{in}, s_{in}, f_{in})$ while the output $y_{tr}$ is of shape $(t_{out}, s_{out}, f_{out}) = (0.5 \times t_{in}, s_{in}, f_{in})$ The cell also receives the decision group $\alpha _{xtr}$ indicating the number of filters in $x_{tr}$.

#### Sensor-fusion cell

**Fig. 6:**
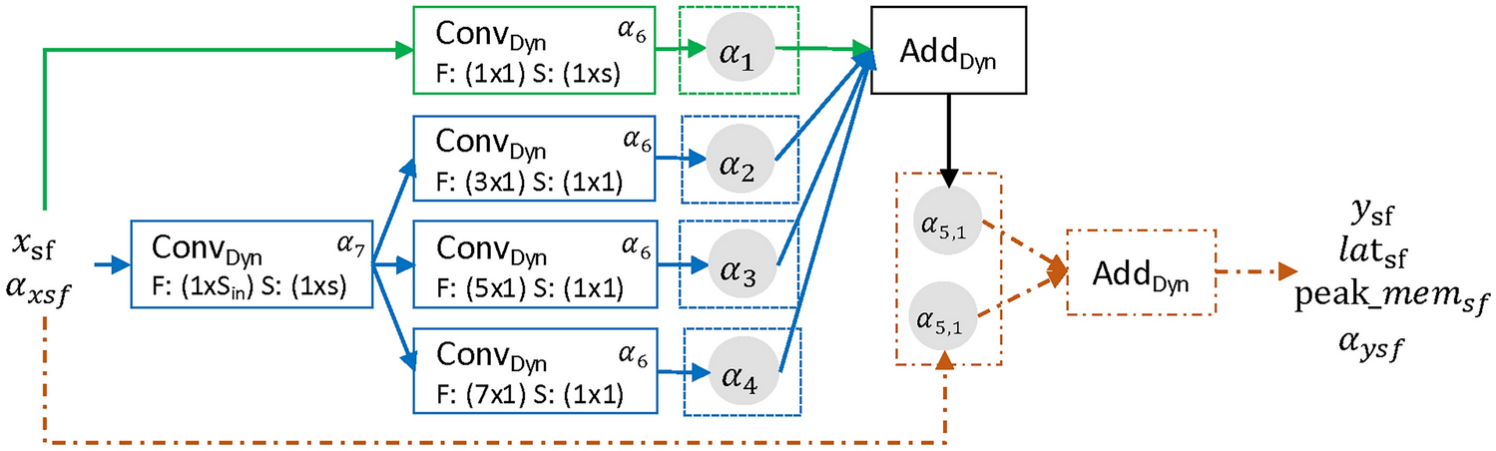
Sensor-Fusion cell. Consists of three pathways, can be configured with stride one or two and depending on that contains six or seven decision groups. *F* denotes the filter size while *S* denotes the stride configuration. The orange pathway is only active, when $stride_{sf} = 1$.

A common problem when dealing with time series data is the interaction between the different sensors. The same phenomena are captured using different sensors, possibly at different positions. The information from these sensors needs to be combined in order to carry useful information^19,43^. To tackle this problem, the Sensor-Fusion cell, inspired by InceptionTime^7^, seen in Fig. 6 was designed. Input to the cell is a time series $x_{sf}$ of shape $(t_{in}, s_{in}, f_{in})$. The cell can be configured with stride $stride_{sf}$ to be equal to 1 or 2 which influences the output shape to be $(t_{in}, s_{in} / stride_{sf}, f_{in})$. When the stride equals one, three pathways through the layer exist, shown in green, blue and orange. The orange pathway (dashed) is an identity connection which can be used to skip the layer and is only included if the input and output of the layer have the same shape and is therefore omitted when the stride equals 2. In the main pathway through the cell (shown in blue), first, a dynamic convolution with filter size $(1, S_{in})$ is applied to allow cross channel interaction by performing convolution across all sensor-channels. Then, in a next step, multiple convolutions with filter sizes $(f,1), \, f \in \{3,5,7\}$ are applied. Each of these convolutions can be individually turned on or off by the search algorithm using the decision groups $\alpha _{2,3,4}.$ This allows features to be extracted simultaneously at different temporal scales if necessary. In Fig. 6, these decision groups are drawn with only one weight, although in reality, for each of the three convolutions, a second parallel zero-connection exists as an alternative which allows the individual selection process. In addition, a skip-connection (shown in green) can be added to the layer using $\alpha _1$. As all the pathways through the cell must output tensors with the same shape, the dynamic convolutions in the skip-connection and the dynamic convolutions in the main-block share their decision group $\alpha _6$ to select the number of filters.

#### Output cell

**Fig. 7:**
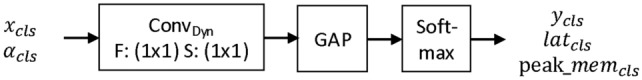
The output cell features a fixed architecture and is therefore not searchable. First, a dynamic convolution is applied to deal with the different number of channels in $x_{cls}$. Finally, global average pooling and a Softmax operation are utilized to output the class probabilities.

The output cell as seen in Fig. 7 has a no learnable architecture. It consists of a dynamic convolution where the number of filters is fixed to the number of classes. Finally, class probabilities ($y_{cls}$) are output using a Global Average Pooling (GAP) and Softmax layer.

## Search algorithm

To search for a suitable architecture in the search space, we apply a modified version of the DNAS algorithm introduced in DARTS^13^. We adapt the algorithm to be hardware aware using a multi-objective loss to optimize the architectural weights $\alpha$. To force the individual decision groups to converge, we employ the Gumbel-Softmax-function^44^ with decreasing temperature $\tau$ as the $pseudo\_prob$-function. Therefore, we optimize the architectural weights $\alpha$ using the loss-function shown in Eq. (6).

6 $$\begin{aligned} \mathscr {L}(\alpha , w, MCU_t, Lat_t, Mem_t) = loss_{val}(\alpha , w) + loss_{lat}(\alpha , MCU_t, Lat_t) + loss_{mem}(\alpha , MCU_t, Mem_t) \end{aligned}$$

In the equation, $loss_{val}$ denotes the cross-entropy loss on the validation dataset. $loss_{lat}$ and $loss_{mem}$ describe the losses caused by the hardware utilization which depend on the search-space configuration $\alpha$, $MCU_t$ and the user defined hardware limits $Lat_t$ and $Mem_t$. The hardware loss functions are formulated as

7 $$\begin{aligned} \begin{aligned} \text {loss}_{\text {lat}}&= \gamma _{\text {lat}} \cdot \log \left( \frac{\text {Lat}(\alpha , \text {MCU}_t)}{\text {Lat}_t}\right) \cdot [\text {Lat}(\alpha , \text {MCU}_t) \ge \text {Lat}_t] \\ \text {loss}_{\text {mem}}&= \gamma _{\text {mem}} \cdot \log \left( \frac{\text {Mem}(\alpha , \text {MCU}_t)}{\text {Mem}_t}\right) \cdot [\text {Mem}(\alpha , \text {MCU}_t) \ge \text {Mem}_t] \end{aligned} \end{aligned}$$

The parameter $\gamma$ weights the importance of the individual loss-terms and needs to be set sufficiently high to ensure the user-defined hardware limits are not violated. The complete search-algorithm can be seen in algorithm 1 where both sets of weights are optimized in an iterative fashion.

**Algorithm 1:**
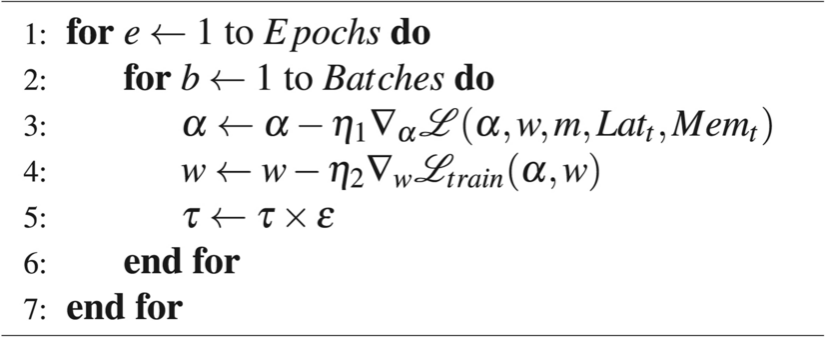
Search algorithm

## Model retraining and quantization

During architecture search, weight sharing between architectures is applied, which allows for an efficient search but at the same time prevents any single architecture to obtain its optimal weights. Therefore, after an architecture has been found, this architecture is trained from scratch to achieve the maximum performance. For this, we use the Adam optimizer for 100 epochs to minimize the negative log likelihood loss. Training is performed in a quantization aware fashion as we later deploy the resulting model to an MCU using $int\_8$ quantization. This greatly reduces computational cost on the microcontroller in terms of execution latency, peak memory consumption and storage requirements with only a minimal loss in classification performance.

## Evaluation

To demonstrate the capabilities of MicroNAS, we evaluate it on two widely-used benchmark datasets from the domain of human activity recognition. The UCI-HAR dataset^45^ consists of a window size of 128, recorded at a sampling rate of 50 Hz, and contains nine sensor channels across six activity classes. In contrast, the SkodaR dataset^46^ has a window size of 64 data points, 30 sensor channels, and was recorded at 96 Hz. The evaluation was conducted on three hardware platforms: The Nucleo-F446RE^47^, featuring a STM32F446RE (ARM Cortex-M4 (Armv7-M), 32-bit, 180 MHz CPU, 512 KB flash, 128 KB SRAM with FPU and DSP), the NUCLEO-L552ZE-Q^48^, which includes an STM32L552ZE (ARM Cortex-M33 (Armv8-M), 32-bit, 80 MHz CPU, 512 KB flash, 256 KB SRAM with FPU and DSP) and the Arduino Portenta H7^49^, which features an STM32H747XI (ARM Cortex-M7 (Armv7E-M), 32-bit, 480 MHz CPU, 2 MB flash, 1 MB SRAM with DP-FPU and DSP).

### Setup

To demonstrate the ease of use of MicroNAS, the same hyperparameters were used for all the experiments. For the convolutions in the Time-Reduce cells, $f_{max}$ was set to 16, and *g* was set to 4. For the Sensor-Fusion cells, $f_{max}$ was set to 64, and *g* to 8. For the number of cells, $ts_{ml}$ was set to 16, $ts_{ms}$ to 5, and $sf_s$ to 2. These settings were chosen to balance the characterization cost of building the latency lookup table and search space flexibility. For the search algorithm, we set $\varepsilon$ to 0.997, $\eta _{lat}$ to 2, and $\eta _{mem}$ to 4. With this setup, the search space for the UCI-HAR dataset contains approximately $10^{13}$ architectures and approximately $10^{22}$ for the SkodaR dataset. We search for 100 epochs with a batch size of 32. To optimize the weights *w*, we use the negative log likelihood and the Adam optimizer. For the architectural weights $\alpha$, we employ our custom loss, as described in Eq. (6), which is optimized by stochastic gradient descent. Here, the learning rate is set to 0.36. We executed each experiment three times and report the mean performance metrics in the plots. Training on an Intel Core i7 12700k takes about 50 minutes for the UCI-HAR dataset and about 1 hour and 15 minutes for the SkodaR dataset.

### MicroNAS under different computational resource constraints

#### Latency versus performance

**Fig. 8:**
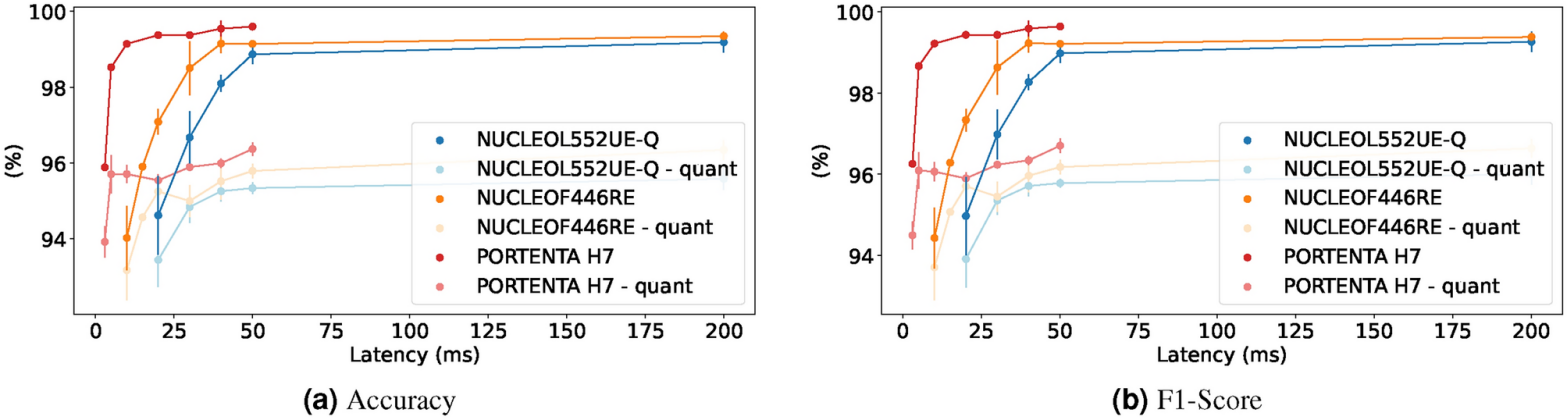
Latency trade-offs on the UCI-HAR dataset.

**Fig. 9:**
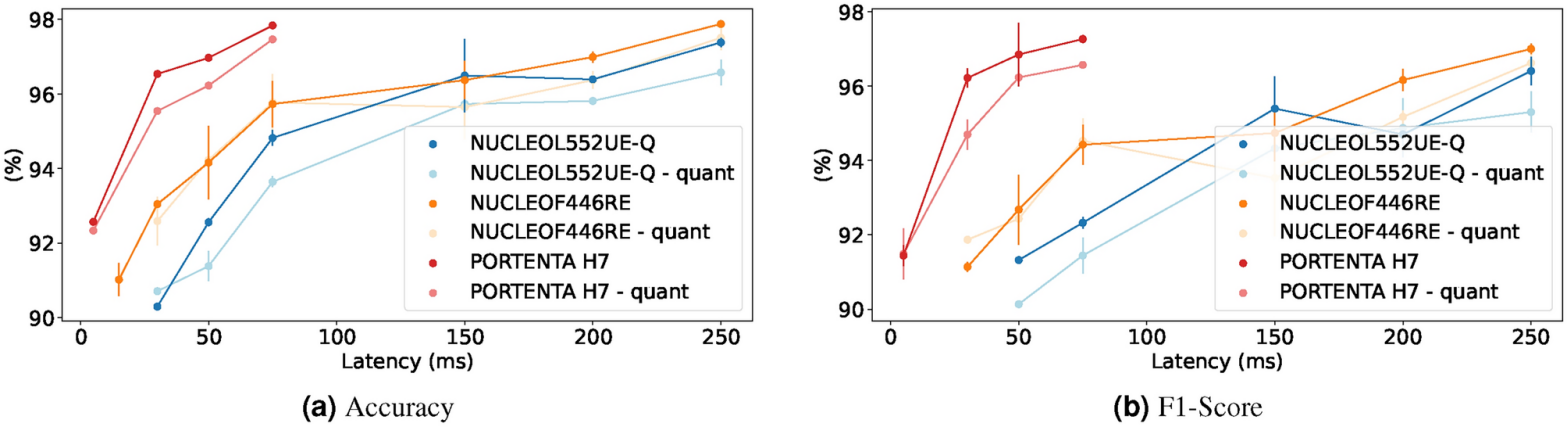
Latency trade-offs on the SkodaR dataset.

**Fig. 10:**
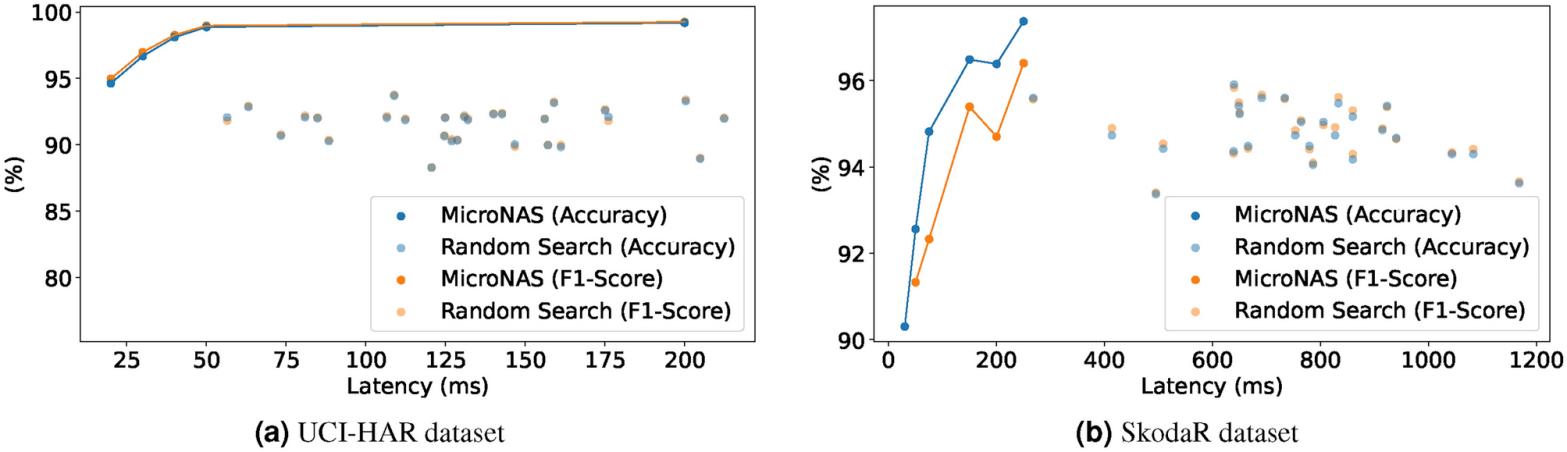
Comparison to random search.

This experiment demonstrates the ability of MicroNAS to discover architectures under varying latency constraints for which we disable the loss caused by the peak-memory consumption. Results can be seen graphically in Fig. 8 for the UCI-HAR dataset^45^ and in Fig. 9 for the SkodaR dataset^46^. Additionally, concrete numbers can be found in Table 1 for the UCI-HAR dataset and in Table 2 for the SkodaR dataset. Generally we can observe that the performance of the models increases with the allowed amount of computation time. When comparing architectures with an allowance of 25 ms on the UCI-HAR dataset, the Nucleo-L552ZE-Q achieves an accuracy of $94.62 \% \pm 1.03 \%$ vs $97.08 \% \pm 0.33 \%$ on the Nucleo-F446RE and $99.37 \%\pm 0.03 \%$ on the Portenta H7. This effect is even more severe on the SkodaR dataset where for an allowed latency of 50 ms, the Nucleo-L552ZE-Q achieves an accuracy of $92.56 \% \pm 0.10 \%$ vs $94.16 \% \pm 0.99 \%$ on the Nucleo-F446RE and $96.92 \%\pm 0.08 \%$ on the Portenta H7. As anticipated, classification accuracy improves with the allowed amount of computation time as seen in Fig. 8 for the UCI-HAR dataset^45^ and Fig. 9 for the SkodaR dataset^46^. Furthermore, we observe that lower latency targets significantly degrade performance on the Nucleo-L552ZE-Q, likely due to its less performant CPU in comparison to the Arduino Portenta or the Nucleo-F446RE (Fig. 10).

**Fig. 11:**
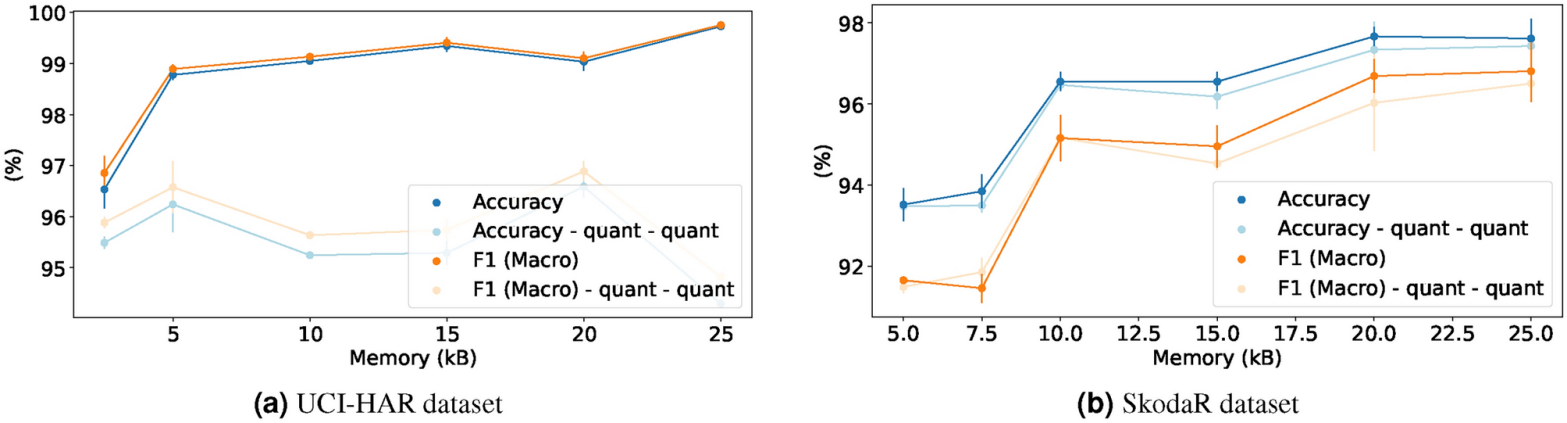
Trade-off between peak memory consumption and Accuracy/F1-Score (Macro).

#### Peak memory versus performance

This experiment demonstrates the ability of MicroNAS to find architectures under different peak memory constraints, where we disable the loss caused by the execution latency. As the TFLM framework^50^ is using the same amount of memory on every microcontroller, this experiment is independent of the microcontroller. We execute each experiment three times and report mean performance metrics, shown in Fig. 11. We expect performance to increase as more memory is allowed to be used which can be observed in the graphic. For the SkodaR dataset (Fig. 11b), this effect is more pronounced than for the UCI-HAR dataset (Fig. 11a). For both datasets it can be seen that performance is relatively constant as long as enough peak memory is allowed. When not enough peak memory is allowed (less then 5kB for the UCI-HAR dataset and less then 10kB for the SkodaR dataset), performance drops significantly.

**Table 1:** Three architectures found by MicroNAS for the UCI-HAR dataset compared to SOTA classifiers^19,25,51,52^.

Model	Device	#Params	Latency (ms)	Peak Memory (B)	Accuracy non-quant (%)	Accuracy quant (%)	F1-Score non-quant (%)	F1-Score quant (%)
MicroNAS 1	NUCLEO	2019.33	19.00	3504.00	$94.62 \pm 1.03$	$93.44 \pm 0.71$	$94.97 \pm 0.96$	$93.91 \pm 0.69$
MicroNAS 2	NUCLEO	43976.67	163.00	14997.33	$99.18 \pm 0.27$	$95.58 \pm 0.30$	$99.26 \pm 0.24$	$96.00 \pm 0.27$
MicroNAS 3	NUCLEOF446RE	998.00	10.00	5856.00	$94.02 \pm 0.85$	$93.17 \pm 0.79$	$94.43 \pm 0.74$	$93.71 \pm 0.81$
MicroNAS 4	NUCLEOF446RE	68790.00	127.00	15360.00	$99.34 \pm 0.12$	$96.34 \pm 0.26$	$99.37 \pm 0.11$	$96.64 \pm 0.22$
MicroNAS 5	PORTENTA	3340.00	2.00	4044.00	$95.88 \pm 0.07$	$93.92 \pm 0.41$	$96.26 \pm 0.06$	$94.50 \pm 0.34$
MicroNAS 6	PORTENTA	114608.00	40.75	16800.00	$99.60 \pm 0.04$	$96.37 \pm 0.18$	$99.63 \pm 0.04$	$96.70 \pm 0.17$
Kolkar et al.	Desktop	NA*	NA*	NA*	96.83	NA*	NA*	NA*
Dua et al.	Desktop	NA*	NA*	NA*	96.20	NA*	96.19	NA*
TinyHar	Desktop	71532	ND*	ND*	99.18	ND*	99.20	ND*
MLP-HAR	Desktop	41924	ND*	ND*	95.65	ND*	95.96	ND*
SVC	Desktop	–	–	–	77.72	–	74.21	–
RF	Desktop	–	–	–	95.11	–	95.10	–
DT	Desktop	–	–	–	95.65	–	95.36	–
LR	Desktop	–	–	–	82.34	–	81.51	–
DARTS-softmax	Nucleo-L552ZE-Q	80098	212.28	18492	95.08	93.45	95.15	93.54
DARTS-gumbel	Nucleo-L552ZE-Q	56390	337.52	20480	93.99	92.35	94.1	92.27

NA* Data not available from the source (experiment from the literature)

ND* Model cannot run on MCUs (self-conducted experiment)

**Table 2:** Three architectures found by MicroNAS for the SkodaR dataset compared to SOTA classifiers^8,19,21,25^.

Model	Device	#Params	Latency (ms)	Peak memory (B)	Accuracy non-quant (%)	Accuracy quant (%)	F1-Score non-quant (%)	F1-Score quant (%)
MicroNAS 1	NUCLEO	4602.00	48.00	8640.00	$90.30 \pm 0.00$	$90.71 \pm 0.00$	$91.32 \pm 0.03$	$90.14 \pm 0.06$
MicroNAS 2	NUCLEO	31258.00	225.00	24576.00	$96.39 \pm 0.06$	$95.81 \pm 0.03$	$96.41 \pm 0.38$	$95.30 \pm 0.55$
MicroNAS 3	NUCLEOF446RE	5678.00	28.00	5840.00	$91.02 \pm 0.44$	$92.59 \pm 0.64$	$91.14 \pm 0.14$	$91.87 \pm 0.05$
MicroNAS 4	NUCLEOF446RE	50018.00	189.00	28800.00	$96.99 \pm 0.15$	$97.50 \pm 0.32$	$97.00 \pm 0.14$	$96.62 \pm 0.50$
MicroNAS 5	PORTENTA	3567.00	4.25	7248.00	$92.76 \pm 0.32$	$92.46 \pm 0.21$	$91.44 \pm 0.28$	$91.49 \pm 0.69$
MicroNAS 6	PORTENTA	81675.00	60.50	33120.00	$97.93 \pm 0.16$	$97.52 \pm 0.11$	$97.27 \pm 0.11$	$96.57 \pm 0.03$
DeepConvLSTM	Desktop	1.1M	NA*	NA*	NA*	NA*	98.99	NA*
Mahmud et al.	Desktop	NA*	NA*	NA*	NA*	NA*	97	NA*
TinyHar	Desktop	114540	ND*	ND*	98.45	ND*	98.27	ND*
MLP-HAR	Desktop	175054	ND*	ND*	94.74	ND*	93.11	ND*
SVC	Desktop	–	–	–	83.28	–	77.73	–
RF	Desktop	–	–	–	92.88	–	91.67	–
DT	Desktop	–	–	–	87.62	–	85.58	–
LR	Desktop	–	–	–	89.47	–	87.52	–
DARTS-softmax	Nucleo-L552ZE-Q	126414	1125.76	30768	91.26	90.77	91.26	86.16
DARTS-gumbel	Nucleo-L552ZE-Q	Failed**						

NA* Data not available from the source (experiment from the literature)

ND* Model cannot run on MCUs (self-conducted experiment)

### Comparison to baselines

To better understand the performance of MicroNAS, we compare it against several baselines, including a random-search baseline on our search space, a DARTS-based baseline, and state-of-the-art time series classification systems found in the literature. Results can be found in Table 1 for the UCI-HAR dataset and in Table 2 for the SkodaR dataset.

#### MicroNAS compared against random search

To validate our proposed search algorithm, we compare it against a random search baseline, which is often hard to beat^53^. Therefore, 30 architectures were randomly sampled from the search space and trained using the same retraining parameters as in the other experiments. The training of the 30 architectures takes about the same time as one complete architecture search. For this experiment we utilize the Nucleo-L552ZE-Q. Results can be seen in Fig. 10. In the experiment, MicroNAS is able to find architectures on the Pareto front relative to the random search baseline. The best randomly searched architecture achieves an accuracy of 93.69 % on the UCI-HAR dataset at 108.89 ms latency and 95.91 % on the SkodaR dataset at 639.69 ms. In comparison, on the UCI-HAR dataset, MicroNAS achieves an accuracy of 94.62 % ± 1.03 % when allowed 25 ms and on the SkodaR an accuracy of 94.81 % ± 0.2 % when allowed 75 ms.

### Comparison to the state-of-the-art

To assess the effectiveness of MicroNAS, we conducted comprehensive comparisons against both state-of-the-art time series classification systems and traditional machine learning approaches using the SkodaR^46^ and UCI-HAR^45^ datasets. The results are presented in Tabel 1 and Table 2. Our evaluation shows, that MicroNAS is able to achieve performances close to the state-of-the-art when comparing against time series classification systems found in the literature although these systems are running on desktop computers while neural networks found by MicroNAS are running on MCUs. Furthermore, we compare MicroNAS against traditional classifiers, namely Support Vector Machines (SVC), Random Forests (RF), Decision Trees (DT) and Logistic Regression (LR). We use the scikit-learn library with the default parameters of the classifiers, except for the RF, where we limit the number of trees to 50 and the maximum depth to 10 so that the resulting models, in theory, can fit onto the Nucleo-L552ZE-Q and Nucleo-F446RE MCUs which both offer 512 kB of flash memory. To train the classifiers, we first apply feature extraction using the tsfresh Python library. The results are particularly noteworthy: On the UCI-HAR dataset, MicroNAS outperformed the best traditional approach (Decision Tree classifier at 95.65% accuracy). Similar superior performance was observed on the SkodaR dataset, as detailed in Table 2. These results are especially significant as they were achieved without requiring manual parameter tuning or model selection. Additionally, we evaluated against a DARTS-based baseline, modified to use our search space for time series classification compatibility. We tested this baseline using both the original Softmax implementation and the Gumbel-Softmax function. The results revealed two key limitations of the DARTS-based approach: first, it lacks hardware awareness, leading to models that lack behind MicroNAS ’s performance but consume significantly more resources; second, in one instance, it produced an invalid disconnected neural network architecture, as noted in Table 2. Further comparisons to other HW-NAS-systems are not possible because they do not target the problem of time series classification and therefore have incompatible search spaces.

## Discussion

In comparison to existing systems, MicroNAS is the first to bring time series classification to microcontrollers using neural architecture search in a hardware aware fashion. As many IoT and wearable devices are equipped with a variety of time series producing sensors, whose data must be processed, we expect many application scenarios to benefit from our presented methodology. Especially when user data needs to be processed privately, in real-time or a connection to a server in the cloud is not feasible.

### Limitations and future work

Besides the neural network architecture, sampling rate, window size, and sensor selection can also impact classification performance^54,55^. To create a complete end-to-end time series classification search system for MCUs, the proposed system can be expanded to encompass these parameters in the search space. This expansion is no trivial task, as the optimization of these parameters must be expressed as a differentiable optimization problem to apply the DNAS framework.

## Conclusion

This paper introduced MicroNAS, a first-of-its-kind hardware-aware neural architecture search (HW-NAS) system specifically designed for time series classification on resource-constrained microcontrollers. By utilizing two types of searchable cells, MicroNAS can be used for various datasets that differ in the window-length and the number of sensors. This, coupled with the possibility to set limits on the execution latency and peak memory consumption makes the system usable in various application scenarios such as privacy critical or real-time systems. The used lookup-table latency estimation approach allows to precisely calculate the execution latency of architectures in the search space and therefore enables MicroNAS to be used in real-time systems. Our experimental results indicate, that for a variety of different hardware limits, MicroNAS is able to find a suitable neural network architecture while closely approaching classification performances to that of state-of-the-art desktop models with 99.60% vs. 99.18% accuracy for the UCI-HAR dataset and 97.93% vs. 98.45% accuracy on the SkodaR dataset. In addition, our experiments regarding latency-performance and memory-performance trade-offs highlight the flexibility and adaptability of our approach. On the UCI-HAR dataset, MicroNAS is able to find architectures with execution latencies from 2 to 163 ms and accuracies ranging from 94.62 to 99.60%. On the SkodaR dataset, accuracies ranged from 90.30 to 97.93% for latencies from 4.25 to 225.00 ms.

## Data Availability

The analysis code is available to researchers upon reasonable request. Please contact Tobias King at tobias.king@kit.edu for access.

## References

[1] Cao, K., Liu, Y., Meng, G. & Sun, Q. An overview on edge computing research. *IEEE Access* **8**, 85714–85728. 10.1109/ACCESS.2020.2991734 (2020).

[2] Rashid, N., Demirel, B. U. & Abdullah-Al-Faruque, M. AHAR: Adaptive CNN for energy-efficient human activity recognition in low-power edge devices. *IEEE IoT J.* **9**, 13041–13051. 10.1109/JIOT.2022.3140465 (2022).

[3] Abbas, N., Zhang, Y., Taherkordi, A. & Skeie, T. Mobile edge computing: A survey. *IEEE IoT J.* **5**, 450–465. 10.1109/JIOT.2017.2750180 (2018).

[4] Chen, J. & Ran, X. Deep learning with edge computing: A review. *Proc. IEEE* **107**, 1655–1674. 10.1109/JPROC.2019.2921977 (2019).

[5] Sehrawat, D. & Gill, N. S. Smart sensors: Analysis of different types of IoT sensors. In *2019 3rd International Conference on Trends in Electronics and Informatics (ICOEI)* 523–528. 10.1109/ICOEI.2019.8862778 (2019).

[6] Zhang, Z., Wang, L. & Lee, C. Recent advances in artificial intelligence sensors. *Adv. Sens. Res.* **2**, 2200072. 10.1002/adsr.202200072 (2023).

[7] Ismail Fawaz, H. et al. Inceptiontime: Finding alexnet for time series classification.. *Data Min. Knowl. Discov.* **34**, 1936–1962. 10.1007/s10618-020-00710-y (2020).

[8] Ordóñez, F. J. & Roggen, D. Deep convolutional and lstm recurrent neural networks for multimodal wearable activity recognition. *Sensors*[SPACE]10.3390/s16010115 (2016). 26797612 10.3390/s16010115 PMC4732148

[9] Sliwa, B., Piatkowski, N. & Wietfeld, C. LIMITS: Lightweight machine learning for IoT systems with resource limitations. In *ICC 2020—2020 IEEE International Conference on Communications (ICC)* 1–7. 10.1109/ICC40277.2020.9149180 (2020).

[10] Watanabe, D. et al. An architectural study for inference coprocessor core at the edge in IoT sensing. In *2020 2nd IEEE International Conference on Artificial Intelligence Circuits and Systems (AICAS)* 305–309. 10.1109/AICAS48895.2020.9073992 (2020).

[11] Ghibellini, A., Bononi, L. & Di Felice, M. Intelligence at the IoT edge: Activity recognition with low-power microcontrollers and convolutional neural networks. In *2022 IEEE 19th Annual Consumer Communications & Networking Conference (CCNC)* 707–710. 10.1109/CCNC49033.2022.9700665 (2022).

[12] Mendoza, H., Klein, A., Feurer, M., Springenberg, J. T. & Hutter, F. Towards automatically-tuned neural networks. In *Proceedings of the Workshop on Automatic Machine Learning, Proceedings of Machine Learning Research*, Vol. 64 (eds Hutter, F., Kotthoff, L. & Vanschoren, J.) 58–65 (PMLR, New York, 2016).

[13] Liu, H., Simonyan, K. & Yang, Y. Darts: Differentiable architecture search (2019). arXiv:1806.09055.

[14] Wu, B. et al. Fbnet: Hardware-aware efficient convnet design via differentiable neural architecture search. In *2019 IEEE/CVF Conference on Computer Vision and Pattern Recognition (CVPR)* 10726–10734. 10.1109/CVPR.2019.01099 (2019).

[15] Wan, A. et al. Fbnetv2: Differentiable neural architecture search for spatial and channel dimensions. In *2020 IEEE/CVF Conference on Computer Vision and Pattern Recognition (CVPR)* 12962–12971. 10.1109/CVPR42600.2020.01298 (2020).

[16] Zhang, S. & Zhou, X. Micronet: Realizing micro neural network via binarizing ghostnet. In |it 2021 6th International Conference on Intelligent Computing and Signal Processing (ICSP) 1340–1343. 10.1109/ICSP51882.2021.9408972 (2021).

[17] Liberis, E., Dudziak, L. & Lane, N. D. nas: Constrained neural architecture search for microcontrollers. In *Proceedings of the 1st Workshop on Machine Learning and Systems, EuroMLSys ’21* 70–79 (Association for Computing Machinery, 2021). 10.1145/3437984.3458836.

[18] Cai, H., Zhu, L. & Han, S. Proxylessnas: Direct neural architecture search on target task and hardware (2019). arXiv:1812.00332.

[19] Zhou, Y. et al. Tinyhar: A lightweight deep learning model designed for human activity recognition. In *Proceedings of the 2022 ACM International Symposium on Wearable Computers, ISWC ’22* 89–93 (Association for Computing Machinery, 2022). 10.1145/3544794.3558467.

[20] Cui, Z., Chen, W. & Chen, Y. Multi-scale convolutional neural networks for time series classification. 10.48550/ARXIV.1603.06995 (2016).

[21] Mahmud, S. et al. Human activity recognition from wearable sensor data using self-attention. In *ECAI 2020—24th European Conference on Artificial Intelligence, 29 August–8 September 2020, Santiago de Compostela, Spain* (2020).

[22] Dennis, D. et al. Shallow rnn: Accurate time-series classification on resource constrained devices. In *Advances in Neural Information Processing Systems*, Vol. 32 (Curran Associates, Inc., 2019).

[23] Yang, F. & Zhang, L. Real-time human activity classification by accelerometer embedded wearable devices. In *2017 4th International Conference on Systems and Informatics (ICSAI)* 469–473. 10.1109/ICSAI.2017.8248338 (2017).

[24] Fedorov, I. et al. Tinylstms: Efficient neural speech enhancement for hearing aids. In *Interspeech 2020, interspeech_2020* (ISCA, 2020). 10.21437/interspeech.2020-1864.

[25] Zhou, Y. et al. Mlp-har: Boosting performance and efficiency of har models on edge devices with purely fully connected layers. In *Proceedings of the 2024 ACM International Symposium on Wearable Computers, ISWC ’24* 133–139 (Association for Computing Machinery, 2024). 10.1145/3675095.3676624.

[26] Zoph, B. & Le, Q. V. Neural architecture search with reinforcement learning (2017). arXiv:1611.01578.

[27] Pham, H., Guan, M., Zoph, B., Le, Q. & Dean, J. Efficient neural architecture search via parameters sharing. In *Proceedings of the 35th International Conference on Machine Learning, Proceedings of Machine Learning Research*, Vol. 80 (eds Dy, J. & Krause, A.) 4095–4104 (PMLR, 2018).

[28] Brock, A., Lim, T., Ritchie, J. M. & Weston, N. SMASH: One-shot model architecture search through hypernetworks. CoRR. arXiv:abs/1708.05344 (2017).

[29] Abadade, Y. et al. A comprehensive survey on tinyml. *IEEE Access* **11**, 96892–96922. 10.1109/ACCESS.2023.3294111 (2023).

[30] LightNAS: On Lightweight and Scalable Neural Architecture Search for Embedded Platforms | IEEE Journals & Magazine | IEEE Xplore.

[31] Saha, S. S. et al. Tinyns: Platform-aware neurosymbolic auto tiny machine learning. *ACM Trans. Embed. Comput. Syst.*[SPACE]10.1145/3603171 (2024). 38933471 10.1145/3603171 PMC11200268

[32] Burrello, A. et al. Enhancing neural architecture search with multiple hardware constraints for deep learning model deployment on tiny iot devices. *IEEE Trans. Emerg. Top. Comput.* **12**, 780–794. 10.1109/TETC.2023.3322033 (2024).

[33] Benmeziane, H. et al. Hardware-aware neural architecture search: Survey and taxonomy. In *Proceedings of the Thirtieth International Joint Conference on Artificial Intelligence, IJCAI-21* (ed Zhou, Z.-H.) 4322–4329 (International Joint Conferences on Artificial Intelligence Organization, 2021). 10.24963/ijcai.2021/592. Survey Track.

[34] Liu, P., Wu, B., Ma, H. & Seok, M. Memnas: Memory-efficient neural architecture search with grow-trim learning. In *Proceedings of the IEEE/CVF Conference on Computer Vision and Pattern Recognition (CVPR)* (2020).

[35] Groh, R. & Kist, A. M. End-to-end evolutionary neural architecture search for microcontroller units. In *2023 IEEE International Conference on Omni-layer Intelligent Systems (COINS)* 1–7. 10.1109/COINS57856.2023.10189194.

[36] Lai, L., Suda, N. & Chandra, V. Not all ops are created equal!. 10.48550/ARXIV.1801.04326 (2018).

[37] Qiao, Y., Xu, H., Zhang, Y. & Huang, S. MONAS: Efficient zero-shot neural architecture search for MCUs. 10.48550/arXiv.2408.15034 (2024). arXiv:2408.15034.

[38] Rakhshani, H. et al. Neural architecture search for time series classification. In *2020 International Joint Conference on Neural Networks (IJCNN)* 1–8. 10.1109/IJCNN48605.2020.9206721 (2020).

[39] Chen, D. et al. Scale-aware neural architecture search for multivariate time series forecasting. 10.48550/ARXIV.2112.07459 (2021).

[40] Liberis, E. & Lane, N. D. Differentiable neural network pruning to enable smart applications on microcontrollers. *Proc. ACM Interact. Mob. Wearable Ubiquitous Technol.*[SPACE]10.1145/3569468 (2023).

[41] Liu, C.-L., Hsaio, W.-H. & Tu, Y.-C. Time series classification with multivariate convolutional neural network. *IEEE Trans. Ind. Electron.* **66**, 4788–4797. 10.1109/TIE.2018.2864702 (2019).

[42] Abedin, A., Ehsanpour, M., Shi, Q., Rezatofighi, H. & Ranasinghe, D. C. Attend and discriminate: Beyond the state-of-the-art for human activity recognition using wearable sensors. *Proc. ACM Interact. Mob. Wearable Ubiquitous Technol.*[SPACE]10.1145/3448083 (2021).

[43] Bagnall, A., Lines, J., Bostrom, A., Large, J. & Keogh, E. The great time series classification bake off: A review and experimental evaluation of recent algorithmic advances. *Data Min. Knowl. Discov.* **31**, 606–660. 10.1007/s10618-016-0483-9 (2017). 30930678 10.1007/s10618-016-0483-9 PMC6404674

[44] Jang, E., Gu, S. & Poole, B. Categorical reparameterization with gumbel-softmax. In *5th International Conference on Learning Representations, ICLR 2017, Toulon, France, April 24–26, 2017, Conference Track Proceedings* (OpenReview.net, 2017).

[45] Reyes-Ortiz, J.-L., Oneto, L., SamÃ, A., Parra, X. & Anguita, D. Transition-aware human activity recognition using smartphones. *Neurocomputing* **171**, 754–767. 10.1016/j.neucom.2015.07.085 (2016).

[46] Zappi, P., Roggen, D., Farella, E., Troester, G. & Benini, L. Network-level power-performance trade-off in wearable activity recognition: A dynamic sensor selection approach. *ACM Trans. Embed. Comput. Syst.* **11**, 68:1-68:30. 10.1145/2345770.2345781 (2012).

[47] Microelectronics, S. Nucleo-f446re - stm32 nucleo-64 development board with stm32f446re mcu, supports arduino and st morpho connectivity - stmicroelectronics. https://www.st.com/en/evaluation-tools/nucleo-f446re.html (2023). (Accessed on 08/14/2023).

[48] STMicroelectronics. Nucleo-l552ze-q - stm32 nucleo-144 development board. https://www.st.com/en/evaluation-tools/nucleo-l552ze-q.html (2023). (Accessed on 05/31/2022).

[49] Arduino. Portenta h7–arduino official store (2025). [Online; accessed 2025-02-03].

[50] David, R. et al. Tensorflow lite micro: Embedded machine learning for tinyml systems. In *Proceedings of Machine Learning and Systems*, vol. 3 (eds Smola, A., Dimakis, A. & Stoica, I.) 800–811 (2021).

[51] Kolkar, R. & Geetha, V. Human activity recognition in smart home using deep learning techniques. In *2021 13th International Conference on Information & Communication Technology and System (ICTS)* 230–234. 10.1109/ICTS52701.2021.9609044 (2021).

[52] Dua, N., Singh, S. N. & Semwal, V. B. Multi-input cnn-gru based human activity recognition using wearable sensors. *Computing* **103**, 1461–1478. 10.1007/s00607-021-00928-8 (2021).

[53] Bergstra, J. & Bengio, Y. Random search for hyper-parameter optimization. *J. Mach. Learn. Res.* **13**, 281–305 (2012).

[54] Kim, T. et al. Effects of sampling rate and window length on motion recognition using semg armband module. *Int. J. Precis. Eng. Manuf.* **22**, 1401–1411. 10.1007/s12541-021-00546-6 (2021).

[55] Banos, O., Galvez, J.-M., Damas, M., Pomares, H. & Rojas, I. Window size impact in human activity recognition. *Sensors* **14**, 6474–6499. 10.3390/s140406474 (2014). 24721766 10.3390/s140406474 PMC4029702

